# Gastric adenocarcinoma complicated by an intra‐abdominal abscess: A case report

**DOI:** 10.1002/ccr3.7868

**Published:** 2023-09-11

**Authors:** Zubir S. Rentiya, Carlo Kristian Chu Carredo, Oluwasayo Owolabi, Pugazhendi Inban, Faiza Arslan, Victor Ameh Odoma, Abiodun Adegbite, Maya Ann Francis, Isioma R. Okobia, Taha Sajjad, Aadil Mahmood Khan, Anasonye Emmanuel Kelechi

**Affiliations:** ^1^ University of Virginia Department of Radiation Oncology & Radiology Charlottesville Virginia USA; ^2^ MedStar Georgetown University Hospital Department of Surgery Washington District of Columbia USA; ^3^ Cebu Institute of Medicine Cebu Philippines; ^4^ Lugansk State Medical University Luhansk Ukraine; ^5^ Government Medical College Omandura Chennai India; ^6^ Rawalpindi Medical University (RMC) Rawalpindi Pakistan; ^7^ Indiana University Health Bloomington Indiana USA; ^8^ University of Ibadan Oyo Nigeria; ^9^ Windsor University School of Medicine Cayon Saint Kitts and Nevis; ^10^ University of Benin Benin Nigeria; ^11^ Mountain vista medical centre Mesa Arizona USA; ^12^ University of Illinois Chicago Chicago Illinois USA; ^13^ Texila American University Georgetown Guyana

**Keywords:** gastric adenocarcinoma, human epidermal growth factor receptor 2 (HER2), image‐guided drainage, Intra‐abdominal abscesses, neoadjuvant chemotherapy

## Abstract

Perforated gastric adenocarcinoma is a rare and challenging complication of gastric cancer, which can lead to intra‐abdominal abscesses and other complications. Management of perforated gastric adenocarcinoma with an intra‐abdominal abscess requires a multidisciplinary approach, including empiric antibiotic therapy and fluid resuscitation, partial gastrectomy with Roux‐en‐Y reconstruction, and image‐guided drainage. This case report highlights the complex and challenging nature of managing perforated gastric adenocarcinoma with intra‐abdominal abscesses. Prompt recognition and timely intervention are essential for favorable outcomes. Postoperative care and close follow‐up are also important.

## Graphical abstract

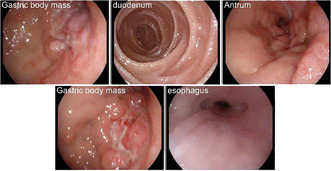

## 1 INTRODUCTION

Perforated gastric adenocarcinoma is a rare but severe complication of gastric cancer, characterized by the rupture of the gastric wall and subsequent leakage of gastric contents into the peritoneal cavity. This condition poses significant diagnostic and therapeutic challenges due to its atypical presentation and potential for intra‐abdominal complications, such as abscess formation.

Gastric adenocarcinoma is the most common type of stomach cancer, accounting for approximately 90% of all cases.^1^ It arises from the glandular cells lining the innermost layer of the stomach and typically presents with nonspecific symptoms, such as abdominal pain, weight loss, and dyspepsia. However, perforation of gastric adenocarcinoma is an uncommon occurrence, with an estimated incidence ranging from 0.3% to 6.4% of all gastric cancer cases.^2^ When perforation occurs, it results in the release of gastric contents, including hydrochloric acid and digestive enzymes, into the peritoneal cavity. This event triggers a cascade of inflammatory responses, leading to the development of an intra‐abdominal abscess.^3^

Intra‐abdominal abscess formation further complicates the clinical course and management of perforated gastric adenocarcinoma, as it necessitates prompt and aggressive intervention to prevent life‐threatening complications such as sepsis.^4^ The management of this rare and complex condition involves a multidisciplinary approach, including surgical intervention, antimicrobial therapy, and supportive care. Prompt diagnosis and timely surgical intervention are crucial to improving patient outcomes, as delayed treatment may lead to increased morbidity and mortality.^5^

In this article, we present a multidisciplinary team approach case of a perforated gastric adenocarcinoma that became complicated by an intra‐abdominal abscess and discuss the challenges encountered in its management. We also review the current literature on the diagnosis, treatment, and outcomes of this uncommon clinical entity, aiming to contribute to the existing knowledge and enhance the understanding of this complex condition with a high risk of death.

## 2 CASE PRESENTATION

We present a case of a delightful 70‐year old woman who initially presented with chief complaints of epigastric pain, unintentional 30‐pound weight loss, and postprandial vomiting for the past 3 months. She reported anorexia and intolerance to solid foods, however, able to tolerate liquids. The patient denied any family history of gastric cancer. However, her sister passed away from pancreatic cancer at the age of 66. Aside from prior resection of her cervix, she was healthy with no medical issues.

On physical examination, the patients' vitals were stable and she was cooperative. The patient had constitutional weight loss, no fevers, no chills, no night sweats, well‐developed, and well‐nourished. She denied any sort of pain. She was alert and oriented, with no acute distress. Her review of systems was unremarkable.

**FIGURE 1:**
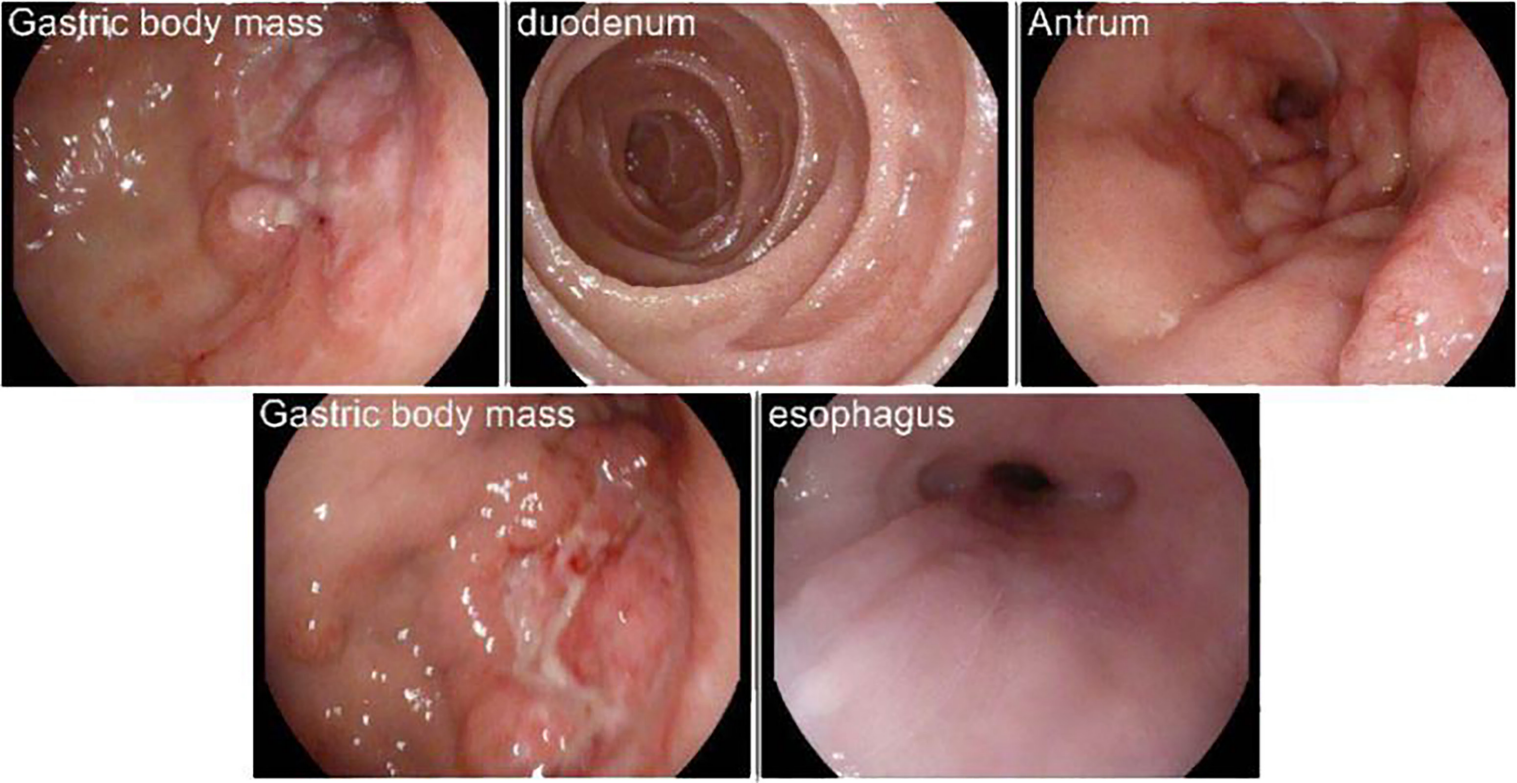
Macroscopic view of a large ulcerated and malignant appearing mass in the gastric body extending into the antrum and pylorus, approximately 10 cm from the gastroesophageal junction.

**FIGURE 2:**
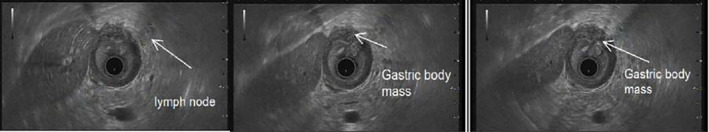
Ultrasound image showing a hypoechoic heterogeneous mass [arrow] in the gastric body and antrum region invading the muscularis propria and serosa, with no apparent invasion of adjacent structures. Additionally, two enlarged adjacent lymph nodes are observed [arrow].

A gastroenterology (GI) ultrasound and endoscopy was performed which showed an ulcer with a fungating diffuse distal gastric mass spanning from the body to the antrum and pylorus highly suspicious for adenocarcinoma as shown in Figures 1 and 2. Pre‐therapy cross‐sectional imaging showed a thickened stomach and a 0.8 × 0.5 nodule in the left lung. The pathology showed an invasive poorly differentiated adenocarcinoma with signet ring features, microsatellite instability/mismatch repair (MMR) intact, human epidermal growth factor receptor 2 (HER2) negative, and programmed death‐ligand 1 (PD‐L1) 3%. She then underwent diagnostic laparoscopy which showed diffuse linitis plastica. Peritoneal washing was negative for malignancy. She was started on modified FOLFOX 6 (a combination of fluorouracil, leucovorin, and oxaliplatin) with nivolumab (an immune checkpoint inhibitor).

**FIGURE 3:**
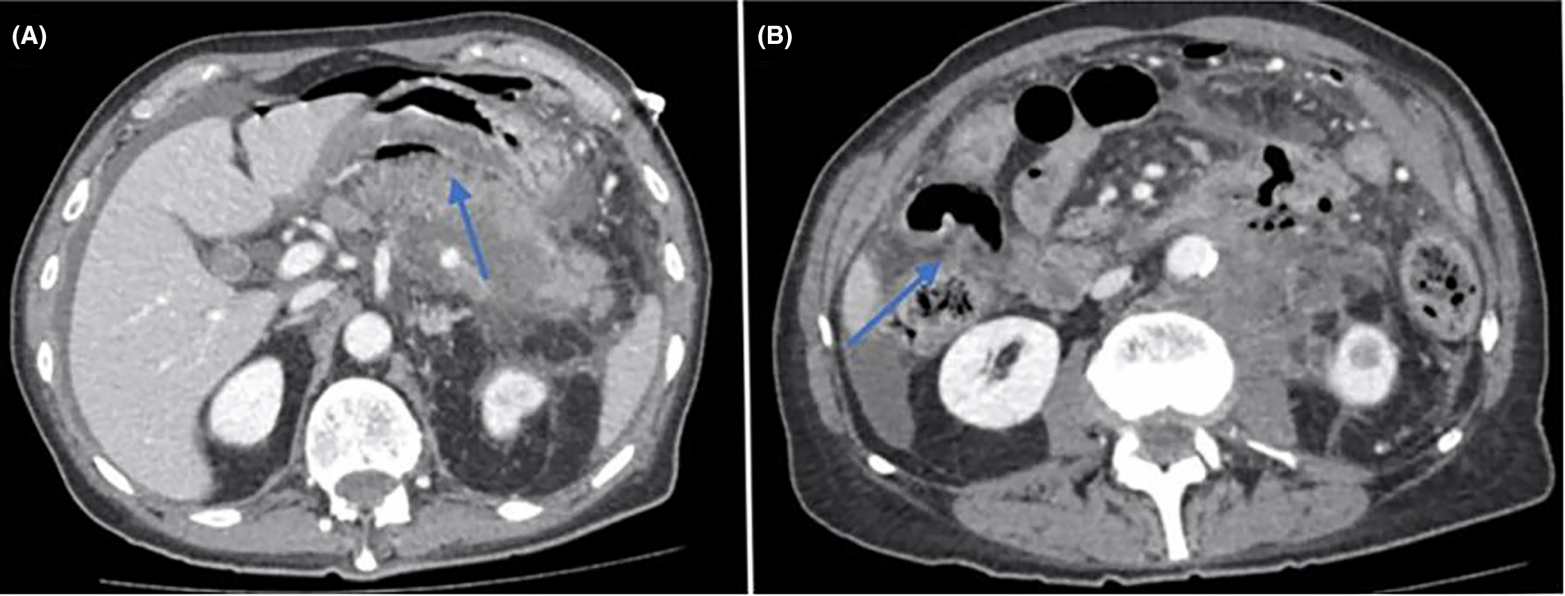
(A) Small bowel perforation involving the anterior aspect of the proximal jejunum [arrow] and (B) large luminal defect at the lesser curvature of the stomach [arrow].

After 3 months, the patient presented with abdominal pain, nausea, bilious emesis, and fever. Her daughters were present and reported that she did not tolerate her chemotherapy infusion that day. She was undergoing neoadjuvant chemotherapy 5/7 cycles with modified FOLFOX 6 with Nivolumab and complained of worsening abdominal pain. The patient was febrile and tachycardic with a white blood cell count of 10.9 with a left shift, normal lactate, and non‐peritonitis on examination. Computed tomography (CT) scan was concerning for contained gastric perforation with a 4 cm fluid collection from the antrum and pylorus in the subhepatic space. CT of the abdomen and pelvis with intravenous contrast showed a gastric antro‐pyloric mass‐like wall thickening with necrosis, localized perforation, and associated complex fluid gas‐containing collection/abscess and regional inflammatory changes as shown in Figure 3.

The patient was started on empiric antibiotic therapy and fluid resuscitation with a resolution of fever and tachycardia. The patient was otherwise being managed conservatively with bowel rest, a nasogastric tube (NGT), and total parenteral nutrition (TPN) via a peripherally inserted central catheter (PICC) and remained hemodynamically stable with a benign exam. She went for partial gastrectomy with Roux‐en‐Y reconstruction. Postoperatively she did well.

**FIGURE 4:**
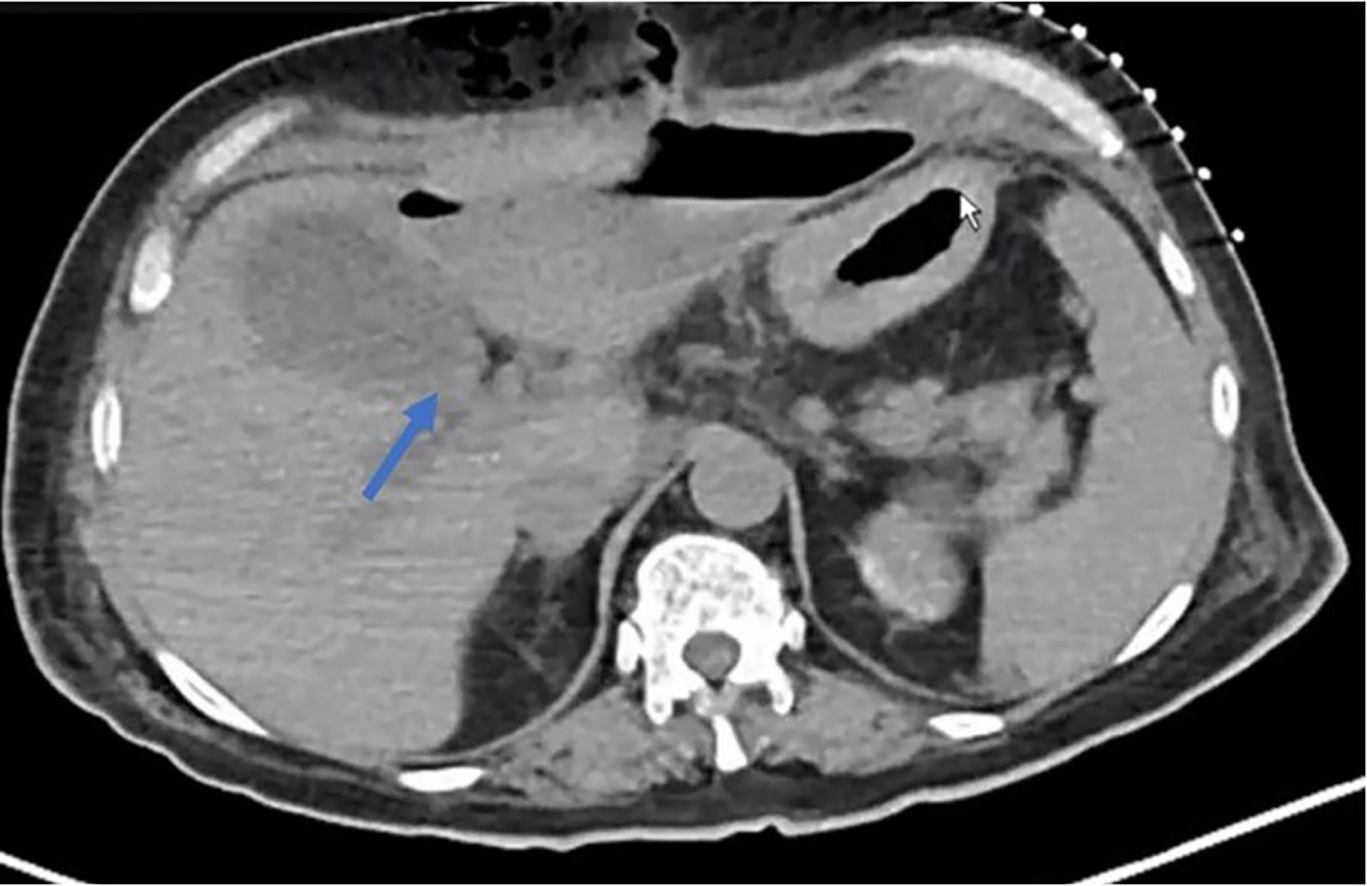
Left hepatic lobe hypoattenuating lesion surrounding the intrahepatic extension of the peri‐gastric fluid gas‐containing accumulation [arrow].

Four weeks later, she was febrile and tachycardic, however, the infectious workup was negative. A repeat CT was done which showed dense right upper quadrant fluid that may reflect a component of hemorrhage and partially walled off right upper quadrant collection in addition to several peri‐gastric lymph nodes as shown in Figure 4. She was managed conservatively with reinitiation of TPN, and her Jackson‐Pratt (JP) drains became bloody appearing. Her subcutaneous heparin was held and she was transfused 2 units of blood. Drain cultures were growing Klebsiella and she completed a 10‐day course of vancomycin, piperacillin, tazobactam, and was stable for discharge.

The family reported foul drainage in the JP drain and weakness after being discharged from the hospital 3 days after. At this time, she had a temperature of 38°C, the heart rate of 128 beats/minute and WBC of 10. She was started on a vancomycin, piperacillin and tazobactam. CT scan of the abdomen and pelvis showed an intra‐abdominal fluid collection. Interventional radiology was sought out and the patient underwent image‐guided drainage of the abscess. Drain evaluation demonstrated residual collection in the left upper quadrant of the abdomen. No fistulous communication was seen to the bowel. The drain was exchanged and repositioned into the residual collection with the placement of a 10.2 French Multipurpose Drain (MPD). Ultrasound evaluation of the midline abdominal subcutaneous collection demonstrated gas and phlegmonous changes with <1 cc fluid aspirated and sent for culture. The drain culture grew Klebsiella, Streptococcus Viridans, Candida Albicans, and ampicillin‐sensitive Enterococcus. She was clinically improving on inpatient antibiotics and tolerating a diet. The drain was removed on the day of discharge due to no more evident output for days. The patient was discharged with home TPN, nursing, and rehabilitation.

## 3 DISCUSSION

Perforated gastric adenocarcinoma is a rare and challenging complication of gastric cancer. It occurs when there is a breach in the gastric wall, leading to the release of gastric contents into the peritoneal cavity. This can result in the formation of intra‐abdominal abscesses and other complications, further complicating the management of an already aggressive malignancy.^6^

In the presented case, a 70‐year‐old woman presented with symptoms of epigastric pain, weight loss, and postprandial vomiting, which raised suspicion for underlying gastric pathology. The patient's age, constitutional weight loss, and anorexia were concerning for a possible malignant etiology. Furthermore, the presence of a gastric mass on endoscopy, along with radiological findings of thickening and nodules in the stomach and lung, supported the diagnosis of gastric adenocarcinoma. The pathology report revealed invasive poorly differentiated adenocarcinoma with signet ring features, indicating an aggressive subtype of gastric cancer. Molecular testing demonstrated intact MMR, HER2 negativity, and PD‐L1 expression of 3%. These findings have implications for treatment decisions, as they help guide the selection of targeted therapies and immunotherapy.^7^ Neoadjuvant chemotherapy with modified FOLFOX 6 and nivolumab was initiated to downsize the tumor and potentially improve surgical outcomes. However, during treatment, the patient presented with worsening abdominal pain, nausea, bilious emesis, and fever. Imaging studies revealed a contained gastric perforation and an associated abscess formation.

Management of perforated gastric adenocarcinoma with intra‐abdominal abscess requires a multidisciplinary approach. In this case, the patient initially received empiric antibiotic therapy and fluid resuscitation to stabilize her condition. Subsequently, she underwent partial gastrectomy with Roux‐en‐Y reconstruction to address the perforation and remove the tumor. The patient had a favorable postoperative course but later developed a partially walled‐off collection and evidence of hemorrhage, which required conservative management with supportive measures, including TPN and blood transfusion. The patient's clinical course was further complicated by the development of a subcutaneous collection and persistent drainage from a surgical drain after discharge. Image‐guided drainage of the peri‐gastric abscess was safe and effective, offering technical advantages such as excellent visualization of subphrenic space location, direct needle passage through gastric wall, avoiding accidental lung and pleura punctures, iatrogenic injury to interposed vessels, and transcutaneous infection avoidance. No complications developed, allowing for conservative treatment with endoscopic transgastric drainage and antibiotics. As in this case, image‐guided drainage was performed to address the intra‐abdominal fluid collection and cultures from the drain grew multiple organisms, including Klebsiella, Streptococcus Viridans, Candida Albicans, and ampicillin‐sensitive Enterococcus. Intravenous antibiotics were administered, and the patient showed clinical improvement and tolerance to an oral diet. Streptococci are the most prevalent bacterium recovered in cultures of stomach abscess contents, accounting for 75% of cases.^8^ Other pathogens detected include Staphylococci, E. coli, Haemophilus Influenzae, Proteus species, Clostridium Welchii, Pseudomonas Aeruginosa, and Bacillus Subtilis.^9^

The case highlights the complex and challenging nature of managing perforated gastric adenocarcinoma with intra‐abdominal abscesses. Prompt recognition and timely intervention are crucial for favorable outcomes. Surgical resection remains the cornerstone of treatment for localized disease, although, in cases of advanced or metastatic disease, neoadjuvant or palliative chemotherapy are considered.^10^ Intra‐abdominal abscesses often require percutaneous or surgical drainage, accompanied by appropriate antimicrobial therapy. It is important to note that each patient's management should be individualized based on the stage of the disease, performance status, comorbidities, and response to treatment. Regular multidisciplinary tumor board discussions play a crucial role in optimizing patient care, ensuring that treatment decisions are evidence‐based and tailored to the specific needs of the patient. In addition to the management, challenges posed by perforated gastric adenocarcinoma complicated by intra‐abdominal abscesses like in this case, highlight the importance of vigilant postoperative care and close follow‐up. The development of postoperative complications, such as persistent drainage, subcutaneous collections, and secondary infections, emphasizes the need for ongoing monitoring and timely interventions. Collaborative efforts between surgical teams, interventional radiologists, infectious disease specialists, and oncologists are crucial for providing comprehensive care and addressing potential complications promptly.^11^

Continued research and experience in managing such cases will contribute to the development of standardized protocols and improved outcomes for patients with this rare and challenging presentation. The case also underscores the importance of patient and family education regarding the signs and symptoms of postoperative complications. Timely reporting of any concerning symptoms, such as foul drainage or weakness, allows for early intervention and prevents potential complications from progressing. A strong support system and effective communication between the healthcare team and the patient and their family play vital roles in ensuring optimal care and successful recovery.^12^ However, further research is needed to explore the detailed complications associated with rupturing of gastric adenocarcinoma.

## 4 CONCLUSION

Perforated gastric adenocarcinoma is a rare complication of gastric cancer that can lead to the formation of intra‐abdominal abscesses and other complications. Early recognition and timely intervention are crucial for favorable outcomes. Management requires a multidisciplinary approach, tailored to the individual needs of the patient. Surgical resection remains the cornerstone of treatment for localized disease, while neoadjuvant or palliative chemotherapy may be considered in advanced or metastatic disease. Regular multidisciplinary tumor board discussions play a crucial role in optimizing patient care. Vigilant postoperative care and close follow‐up are also essential to address potential complications promptly. Further research is needed to explore the detailed complications associated with rupturing of gastric adenocarcinoma.

## AUTHOR CONTRIBUTIONS

**Zubir S. Rentiya:** Writing – review and editing. **Carlo Kristian Chu Carredo:** Writing – original draft. **Oluwasayo Owolabi:** Writing – original draft. **Pugazhendi Inban:** Writing – original draft. **Faiza Arslan:** Conceptualization. **Victor Ameh Odoma:** Validation; writing – original draft. **Abiodun Adegbite:** Writing – original draft. **Maya Ann Francis:** Writing – review and editing. **Okobia Isioma:** Writing – review and editing. **Taha Sajjad:** Writing – review and editing. **AADIL Mahmood KHAN:** Project administration; supervision. **Anasonye Emmnauel Kelechi:** Formal analysis; resources.

## CONFLICT OF INTEREST STATEMENT

The authors have no conflict of interest to declare.

## CONSENT

Written informed consent was obtained from the patient to publish this report in accordance with the journal's patient consent policy.

## Data Availability

The data that support the findings of this study are available from the corresponding author upon reasonable request.
